# Daily stress reactivity and serotonin transporter gene (5-HTTLPR) variation: internalizing responses to everyday stress as a possible transdiagnostic phenotype

**DOI:** 10.1186/2045-5380-4-2

**Published:** 2014-01-24

**Authors:** Christopher C Conway, George M Slavich, Constance Hammen

**Affiliations:** 1 Department of Psychology, University of California, Los Angeles, Box 951563, Los Angeles, CA 90095-1563, USA; 2 Department of Psychology, Cousins Center for Psychoneuroimmunology, University of California, CA, Los Angeles , USA; 3 Department of Psychiatry and Biobehavioral Sciences, University of California , CA, Los Angeles, USA

**Keywords:** Anxiety, Depression, Daily diary, Gene-environment interaction, Intermediate phenotype, Life events, RDoC, Serotonin transporter gene, Young adulthood

## Abstract

**Background:**

Recent studies examining the interaction between the 5-HTTLPR locus in the serotonin transporter gene and life stress in predicting depression have yielded equivocal results, leading some researchers to question whether 5-HTTLPR variation indeed regulates depressive responses to stress. Two possible sources of inconsistent data in this literature are imprecise stress assessment methodologies and a restricted focus on depression phenotypes as the outcome of interest, as opposed to transdiagnostic emotional symptoms such as internalizing and externalizing dimensions. The present study aimed to address these critical limitations in prior research by examining how 5-HTTLPR acts in concert with idiographically assessed daily life stress to predict transdiagnostic emotional outcomes.

**Results:**

One hundred and four healthy young adults genotyped for 5-HTTLPR reported on their life stress exposure and internalizing and externalizing experiences for 14 consecutive days. As hypothesized, daily stress levels were associated with severity of internalizing symptoms, but only for 5-HTTLPR S allele carriers. Additional analyses revealed that these interactive effects of 5-HTTLPR and daily life stress on internalizing symptoms extended to both the distress and fear subdomains of internalizing symptoms.

**Conclusions:**

Considered together, these results support the validity of the 5-HTTLPR stress sensitivity hypothesis and suggest for the first time that variation at 5-HTTLPR moderates the effects of daily life stress on broadband symptom profiles.

## Background

The most widely studied topic in gene-environment interaction (G × E) research over the past decade has been the interplay between a polymorphism in the promoter region of the serotonin transporter gene and life stress in predicting risk for depression. A number of studies have either partly or fully replicated the finding that short (S) allele carriers at 5-HTTLPR are more susceptible to depression in the face of stress than long (L) allele homozygotes [1]. Yet, null results have also been reported; indeed, one recent meta-analysis did not find convincing evidence for the 5-HTTLPR G × E phenomenon [2].

The manner in which life stress is assessed has likely contributed to inconsistent findings across G × E studies. One meta-analytic review that evaluated methodological influences on the magnitude of 5-HTTLPR G × E concluded that the 5-HTTLPR genotype moderates the effects of stress on depression, but only when investigator-based procedures (for example, interview measures, inspection of objective records) are used for stress assessment [3]. Additionally, when exposed to standardized emotional or stressful cues in laboratory settings, S allele carriers consistently exhibit potentiated limbic system activation, cortisol responses, and attentional biases to threat-related stimuli compared to L allele homozygotes [4-6]. Together, these observations suggest that the methods researchers use to assess properties of the environment play a critical role in tests of the 5-HTTLPR stress sensitivity hypothesis [7].

Another pressing and unanswered question is whether 5-HTTLPR is relevant just for depression or, alternatively, for a broader range of stress-linked psychopathology. Whereas initial evidence suggested that 5-HTTLPR genotype, in concert with stress, does not influence anxiety [8], other studies have since implicated the S allele in risk for diverse anxiety and depressive symptoms [7]. Further, the S allele’s association with basic neural, endocrine, and cognitive phenotypes that are linked to a broad array of disorders provides indirect evidence for its involvement in multiple psychopathologies [9].

The goal of the present study was to advance this research by examining the relation of 5-HTTLPR variation to a novel social-environmental context: everyday stressful life events. Using a daily diary methodology, we were able to make an idiographic assessment of fluctuations in stress exposure by determining a person’s average stress levels with repeated measurements; evaluate whether the putative stress-sensitizing effects of 5-HTTLPR extend to day-to-day emotional symptoms; and substantially reduce the interval between stress occurrence and report of symptomatic reactions. Based on prior research, we hypothesized that 5-HTTLPR S allele carriers would exhibit stronger internalizing responses to daily life stress than L allele homozygotes.

We also investigated the specificity of 5-HTTLPR-mediated individual differences in stress reactivity by assessing transdiagnostic emotional phenotypes. Drawing on latent variable research on the meta-structure of mental disorders [10], we collected daily reports of fear (underlying panic and the phobias), distress (unipolar depression and generalized anxiety), and externalizing (disinhibited behavior and substance misuse) phenotypes. These transdiagnostic dimensions are theorized to capture homogeneous clinical traits that are more amenable to genetic analysis than categorical psychiatric diagnoses [11]. Given existing evidence of an association between the S allele and heightened emotional reactivity, we hypothesized that S allele carriers would exhibit stronger fear, distress, and externalizing reactions to everyday stressors than L allele homozygotes.

## Methods

### Participants

Participants were 104 undergraduate students enrolled in an introductory psychology course. The sample included 76 women (73.1%) and 28 men (26.9%), with a mean age of 19.64 years (*standard deviation* = 4.61). Forty-seven participants (45.2%) self-identified as Caucasian, 45 (43.3%) as Latino/a, 5 (4.8%) as biracial, 3 (2.9%) as Asian, 1 (1.0%) as Native American, and 3 (3.0%) as ‘other.’

### Procedures

At a baseline interview, participants completed the Young Adult Self Report questionnaire (YASR; [12]) to assess trait levels of internalizing and externalizing symptoms (see below). They also supplied a saliva sample for genotyping procedures. Participants were then provided instructions for the daily online diary. Diary data were collected on the day of the baseline assessment and the following 13 consecutive days during the middle of the fall and winter academic quarters. The 14-day study period was selected so that the number of weekdays (versus weekend days) would be constant across all participants. Participants were asked to complete the diary as late at night as was convenient for them (from 8pm to 2am); diaries could only be submitted if all questionnaire items were completed. Study procedures were approved by the institutional review board of the University of California, Los Angeles, and all participants provided written informed consent prior to participating in the study.

### Measures

#### ***Daily stressful life events***

Daily stressors were assessed with a 16-item inventory composed of items adapted from instruments designed to elicit self-reports of stressful life events relevant to student life (for example, [13,14]). These items queried daily stressors occurring in several life domains, including interpersonal, achievement, financial and health events. Example events included ‘A friendship ended,’ ‘Failed to achieve an important school-related goal,’ ‘Did not have enough money to do something or buy something,’ and ‘Was sick or had a medical issue’ (see Appendix for complete list). Prior research has shown that life events from this inventory are associated with internalizing symptoms both on a daily basis and over several months [14,15]. Participants indicated whether each event occurred over the course of the current day and, if so, how many times it occurred. The total count of stressors was used in analyses to represent daily stress exposure.

#### ***Baseline internalizing and externalizing symptoms***

The YASR consists of 119 items that assess internalizing and externalizing symptoms, as well as other problem behavior dimensions, including somatic complaints and attention problems. Achenbach [12] provided data to support the internal consistency, test-retest reliability, and criterion validity of the YASR internalizing and externalizing scales. In the present sample, Cronbach’s alpha values for the internalizing and externalizing scales were 0.87 and 0.72, respectively.

#### ***Daily symptoms***

Fear, distress, and externalizing symptoms have not been assessed in previous daily process research. Assessment measures for the present study were created by selecting items from existing instruments that have been constructed to distinguish between these symptom domains in between-subjects research. For example, items that had the largest factor loadings on the YASR externalizing subscale, and that were also relevant to college students, were selected to index daily externalizing behavior. Internalizing items were selected from the Inventory of Depression and Anxiety Symptoms [16], which includes scales that are psychometrically derived to distinguish the fear and distress domains. Inventory of Depression and Anxiety Symptoms items that were judged to be most applicable to the college student population were chosen for inclusion on the daily fear and distress scales.

#### ***Daily internalizing symptoms***

Five items were chosen to represent the fear domain (for example, ‘I felt self-conscious knowing that others were watching me’) and six items were chosen to represent the distress domain (for example, ‘I felt inadequate’) (see Appendix for complete list). Participants were prompted to respond on a five-point Likert-type scale according to ‘How much you have felt or experienced things this way today?’ Responses to distress and fear symptom items were summed to represent the total level of daily internalizing symptoms.

#### ***Daily externalizing symptoms***

The same prompt and response format were used to assess daily severity of externalizing symptoms. Nine items (for example, ‘I was mean to others’) were adapted from the YASR to assess the externalizing domain (see Appendix for complete list).

### Genotyping

Saliva samples were collected under researcher observation for DNA analyses using Oragene saliva collection kits (DNA Genotek, Inc, Ottawa, ON, Canada). Genotyping was performed at the University of California, Los Angeles, Genotyping and Sequencing Core. Polymerase chain reaction (PCR) primers were labeled with fluorescent dye, and PCR was performed on Applied Biosystems dual block PCR thermal cyclers (Foster City, CA, USA). Microsatellite genotypes were run on an Applied Biosystems 3730 capillary DNA sequencer and analyzed using the Applied Biosystems GeneMapper software version 4.0. The 5-HTTLPR polymorphism was assayed on an Applied Biosystems GeneMapper 7900HT Fast Real-Time PCR System and analyzed using the Sequence Detection Systems software version 2.3. Each run included two positive control samples (individual 2 in CEPH family 1347; Coriell Institute). Genotype frequencies were LL = 34, SL = 42, SS = 28, and they were in Hardy-Weinberg equilibrium for the entire sample, as well as for Caucasian and Latino subgroups (*χ*^2^(2) <2, *P* >0.10. In accord with prior research [17], the rs25531 locus was assayed using the protocol described by Wray *et al*. [18] and eight L_G_ alleles were reclassified as S alleles.

### Data analyses

Data analyses were conducted within a hierarchical linear modeling (HLM) framework. HLM is ideal for diary studies because observations at multiple time-points are nested within individuals, resulting in dependencies among residuals. G × E hypotheses were investigated using the following HLM functions:

$${\text{INT}}_{t}=\pi_{0}+\pi_{1}\left({\text{STRESS}}_{t}\right)+\pi_{2}\left({\mathrm{INT}}_{t-1}\right)+e_{t}$$

$$\begin{array}{ll} \pi_{0j}= & \beta_{00}+\beta_{01}\left({\text{GENDER}}_{j}\right)+\beta_{02}\left({\text{GENO}1}_{j}\right)\\ & \;+\beta_{03}\left(\text{GENO}2_{j}\right)+u_{0j} \end{array}$$

$$\begin{array}{ll} \pi_{\mathrm{lj}}= & \beta_{10}+\beta_{11}\left({\text{GENDER}}_{j}\right)+\beta_{12}\left(\text{GENO}1_{j}\right)\\ & \;+\beta_{13}\left(\text{GENO}2_{j}\right)+u_{\mathrm{lj}} \end{array}$$

$$\pi_{2j}=\beta_{20}+u_{2j}$$

where INT_*t*_ represents internalizing symptoms on Day *t*, INT_*t-1*_ represents internalizing symptoms on Day *t-1*, STRESS_*t*_ represents the count of stressors on Day *t*, GENO1_*j*_ represents the contrast between SS and LL genotypes, and GENO2_*j*_ represents the contrast between SL and LL genotypes. Gender was controlled by including it as a between-subjects predictor of the overall intercept (*π*_*0*_) on Level 2.

All Level 1 variables were person-mean centered, such that STRESS_*t*_ indicates the difference between the number of stressors occurring on Day *t* for a given participant and this participant’s mean number of daily stressors across all 14 days. Gender and the genotype contrasts were entered uncentered into Level 2 equations.

The cross-level interactions between the 5-HTTLPR contrasts and daily stress were of primary interest in G × E analyses. A likelihood ratio test was performed to evaluate the significance of the simultaneous addition of genotype contrasts into the *π*_*1*_ equation. In a secondary analysis, product terms composed of genotype and gender, and genotype and ethnicity, were added at Level 2 to examine the consistency of G × E effects across these demographic factors.

We expected to observe main effects of daily life stress on same-day symptoms, but not next-day symptoms, given that lagged effects are rarely detected in daily process studies involving non-clinical populations [19]. Nevertheless, we tested for lagged main effects and G × E on an exploratory basis. It is possible that genetically vulnerable individuals experience a more enduring emotional impact of stressful events, although this was not observed in one previous study of 5-HTTLPR stress reactivity and anxious mood [15]. Along these lines, we used effect size estimates from a prior study of 5-HTTLPR and daily stress reactivity (Table 2 in [15]) to compute *post hoc* power. Following Hox’s [20] equations, and setting alpha to the conventional 0.05 level, we found the present study to have *post hoc* power of 0.74, suggesting sufficient statistical power to detect significant results if they exist.

Data were checked preliminarily for outliers and non-normality. Four participants were considered as possible univariate outliers on the internalizing dimension and four on the externalizing dimension (one case was a possible outlier on both), defined as symptom scores that were greater than two standard deviations from the mean (all were above the mean) across all 14 measurements. The pattern and significance of results were unchanged when these cases were removed, and analyses presented below therefore include all cases. Given positive skew across all four outcome variables, significance testing was based on robust standard errors (also known as Huber/White estimators), which do not depend on the normality of errors assumption [20].

## Results

### Descriptive statistics

The average scores on the YASR internalizing and externalizing scales at baseline were 15.76 (*standard deviation* = 7.56) and 7.90 (*standard deviation* = 4.62), respectively. According to Achenbach’s [12] guidelines, 18 participants (17.3%) were in the borderline clinical or clinical range for internalizing, and 4 participants (3.8%) were in the borderline clinical or clinical range for externalizing. The Pearson correlation between internalizing and externalizing scales was 0.29 (*P* <0.01).

Descriptive statistics for the daily variables are presented in Table 1. Averaged across 14 days, all daily symptom scales demonstrated high levels of internal consistency, with Cronbach’s alpha values of 0.91 for internalizing, 0.91 for fear, 0.93 for distress and 0.78 for externalizing. Participants reported approximately 1.27 stressors per day, with school-related events (not involving grade point average) and medical problems occurring most frequently (approximately once every three days), and lost or stolen property events occurring least frequently (approximately once every 100 days). An average of 11.63 out of 14 diaries (83.1%) were completed on time (that is, before 2am the day after they were mailed), a rate comparable to that of previous studies in college student samples [21]. Compliance was not related to genotype or the YASR scales (*P* >0.10), and the pattern and significance of results were unaltered when participants who missed more than three surveys were omitted. Therefore, all results presented below reflect analyses conducted for the full sample.

**Table 1:** Descriptive statistics for daily variables

**Variable**	**Mean**	**Standard deviation**	**Minimum**	**Maximum**
Daily life stressors	1.27	1.07	0.00	5.57
Internalizing symptoms	17.47	5.14	11.00	38.21
Fear symptoms	7.06	2.07	5.00	16.21
Distress symptoms	10.42	3.40	6.00	22.00
Externalizing symptoms	9.98	1.19	9.00	17.79

*Note.* The scale for each symptom ranged from 1 to 5, with 11 items indexing internalizing symptoms and 9 items indexing externalizing symptoms. The scale for each stressor ranged from 0 to 3. Descriptive statistics were computed by first calculating the within-person value for each participant across time-points and then averaging across participants.

### Stress, 5-HTTLPR, and same-day internalizing and externalizing symptoms

We first examined the direct effects of daily life stress on reports of same-day internalizing and externalizing symptoms. Stress on Day *t* was strongly associated with elevations in internalizing symptoms (*b* = 1.35, standard error (*SE)* = 0.19, *P* <0.001) and externalizing symptoms (*b* = 0.20, *SE* = 0.04, *P* <0.001) on Day *t*. As can be seen in Table 2, genotype and gender did not predict daily symptom levels, with the exception of women reporting significantly higher levels of daily distress symptoms. Prior to investigating G × E effects, we examined the association between genotype and daily stress to rule out the possibility of gene-environment correlation. This analysis demonstrated that 5-HTTLPR genotype was unrelated to daily stress exposure (*b* = -0.02, *SE* = 0.24, *P* = 0.95), thus ruling out gene-environment correlation.

**Table 2:** Hierarchical linear models of 5-HTTLPR × daily life stress interactions: genotype coding

	**Internalizing symptoms**_***t***_			**Fear symptoms**_***t***_			**Distress symptoms**_***t***_			**Externalizing symptoms**_***t***_		
**Predictors**	***b***	***SE***	***P***	***b***	***SE***	***P***	***b***	***SE***	***P***	***b***	***SE***	***P***
For overall intercept, *π*_*0*_												
Intercept, *β*_*00*_	16.71	0.86	<0.001	6.63	0.32	<0.001	10.08	0.57	<0.001	10.05	0.39	<0.001
Gender, *β*_*01*_	-1.96	1.11	0.081	-0.35	0.45	0.445	-1.61	0.69	0.021	-0.22	0.23	0.356
SS versus LL, *β*_*03*_	1.46	1.17	0.216	0.75	0.47	0.110	0.71	0.76	0.356	-0.03	0.38	0.921
SL versus LL, *β*_*04*_	1.49	1.11	0.184	0.52	0.43	0.232	0.96	0.73	0.190	0.00	0.39	0.994
For stress_*t*_ slope, *π*_*1*_												
Intercept, *β*_*10*_	0.63	0.23	0.008	0.10	0.08	0.192	0.54	0.17	0.002	0.05	0.08	0.487
Gender, *β*_*11*_	-0.85	0.30	0.006	-0.26	0.11	0.026	-0.58	0.22	0.010	0.09	0.13	0.464
SS versus LL, *β*_*12*_	1.24	0.46	0.008	0.50	0.16	0.002	0.71	0.34	0.040	0.18	0.10	0.083
SL versus LL, *β*_*13*_	1.18	0.36	0.002	0.36	0.13	0.007	0.77	0.28	0.007	0.16	0.10	0.139
For symptom_*t-1*_ slope, *π*_*2*_												
Intercept, *β*_*20*_	0.10	0.03	0.003	0.07	0.04	0.062	0.14	0.03	<0.001	0.01	0.05	0.753

*Note.* For gender, male = 1, female = 0. LL, 5-HTTLPR long allele homozygosity; SL, 5-HTTLPR heterozygosity; SE, standard error; SS, 5-HTTLPR short allele homozygosity.

**Figure 1:**
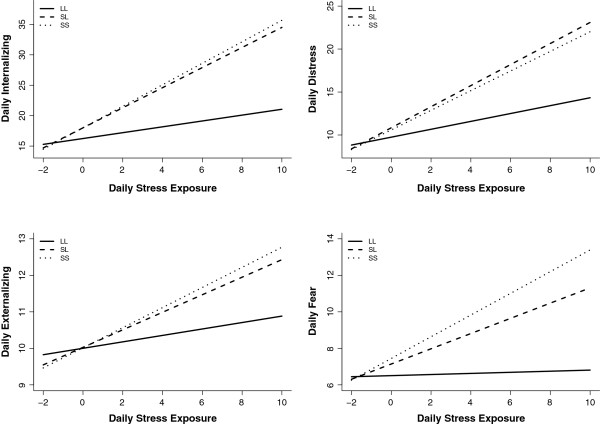
**Within-person associations between daily life stress exposure and transdiagnostic symptoms as a function of 5-HTTLPR genotype.** On the *x*-axis, the count of daily stressors is person-centered, such that 0 represents an average amount of daily stress for a given individual. LL, 5-HTTLPR long allele homozygosity; SL, 5-HTTLPR heterozygosity; SS, 5-HTTLPR short allele homozygosity.

A significant interaction between daily stress and 5-HTTLPR was detected in the prediction of internalizing symptoms (*χ*^2^(2) = 7.77, *P* <0.05). Daily life stress was more strongly related to internalizing symptoms for individuals with the SS and SL genotypes compared to L homozygotes (see genotype contrasts in Table 2). Simple slopes computed for each genotype group revealed that, as hypothesized, the stress-internalizing association was significant for individuals with both the SS genotype (*b* = 1.63, *SE* = 0.34, *P* <0.001) and SL genotype (*b* = 1.52, *SE* = 0.26, *P* <0.001), but not for L homozygotes (*b* = 0.44, *SE* = 0.36, *P* = 0.22). Figure 1 depicts the form of the 5-HTTLPR G × E interaction effect for internalizing symptoms. Regarding the interaction of genotype and daily life stress in predicting daily externalizing symptoms, the omnibus significance test of the two genotype contrasts was not indicative of a G × E effect (*χ*^2^(2) = 2.89, *P* = 0.23), although the comparison between SS and LL group slopes was marginally significant (see Table 2).

Given the close correspondence between SS and SL groups in terms of internalizing and externalizing reactivity to daily stressors (see Figure 1), follow-up analyses were conducted collapsing across SS and SL genotypes to compare the stress-symptom association for S carriers versus L homozygotes. As seen in Table 3, a significant G × E effect was observed for internalizing symptoms, whereby S allele carriers demonstrated greater internalizing reactivity to daily life stress than L homozygotes. Simple effects analyses revealed that daily exposure to stress was associated with higher levels of internalizing symptoms for S carriers (*b* = 1.57, *SE* = 0.21, *P* <0.001) but not L homozygotes (*b* = 0.44, *SE* = 0.36, *P* = 0.22).

**Table 3:** Hierarchical linear models of 5-HTTLPR × daily life stress interactions: allele coding

	**Internalizing symptoms**_***t***_			**Fear symptoms**_***t***_			**Distress symptoms**_***t***_			**Externalizing symptoms**_***t***_		
**Predictors**	***b***	***SE***	***P***	***b***	***SE***	***P***	***b***	***SE***	***P***	***b***	***SE***	***P***
For overall intercept, *π*_*0*_												
Intercept, *β*_*00*_	16.71	0.86	<0.001	6.62	0.31	<0.001	10.09	0.57	<0.001	10.05	0.39	<0.001
Gender, *β*_*01*_	-1.96	1.11	0.079	-0.32	0.44	0.470	-1.63	0.70	0.020	-0.22	0.23	0.345
5-HTTLPR, *β*_*03*_	1.48	0.97	0.132	0.61	0.37	0.109	0.87	0.64	0.178	-0.01	0.37	0.967
For stress_*t*_ slope, *π*_*1*_												
Intercept, *β*_*10*_	0.62	0.23	0.008	0.10	0.08	0.194	0.54	0.17	0.002	0.05	0.08	0.485
Gender, *β*_*11*_	-0.84	0.30	0.006	-0.26	0.12	0.029	-0.59	0.22	0.009	0.09	0.12	0.466
5-HTTLPR, *β*_*12*_	1.20	0.32	<0.001	0.41	0.12	<0.001	0.75	0.24	0.003	0.16	0.09	0.079
For symptom_*t-1*_ slope, *π*_*2*_												
Intercept, *β*_*20*_	0.10	0.03	0.003	0.07	0.04	0.060	0.14	0.03	<0.001	0.01	0.05	0.757

*Note.* For gender, male = 1, female = 0. 5-HTTLPR represents the contrast between S allele carriers and L allele homozygotes, with S allele carriers coded as 1 and LL as 0. SE, standard error.

Analyses also revealed some evidence that daily life stress exposure interacted with genotype to predict externalizing symptoms when the SS and SL genotype groups were combined (*b* = 0.16, *SE* = 0.09, *P* = 0.08). Simple effects analyses revealed that the magnitude of the daily stress-externalizing symptoms association for S carriers (*b* = 0.23, *SE* = 0.05, *P* <0.001) was approximately three times greater than the corresponding association for L homozygotes (*b* = 0.08, *SE* = 0.09, *P* = 0.35).

Consistent with the large literature showing that women are at significantly higher risk for depression relative to men, and that they also exhibit greater internalizing responses to stressful life events [22], women in the present study tended to report greater increases in internalizing symptoms, but not externalizing symptoms, in response to daily life stress (Table 2). However, gender did not moderate the strength of G × E effects for either internalizing or externalizing symptoms (*t* <1.00, *P* >0.10). Additionally, the strength of the G × E effects did not vary across Caucasian versus Latino ethnic groups (which collectively made up approximately 93% of our sample) for either the internalizing (*b* = -0.08, *SE* = 0.57, *P* = 0.88) or externalizing (*b* = -0.02, *SE* = 0.19, *P* = 0.93) dimensions. Likewise, ethnicity was not related to 5-HTTLPR genotype, stress exposure or any daily symptom outcome (*P* >0.10).

### Stress, 5-HTTLPR and fear and distress symptoms

We observed strong effects of stress on both same-day distress symptoms (*b* = 0.98, *SE* = 0.15, *P* <0.001) and fear symptoms (*b* = 0.35, *SE* = 0.06, *P* <0.001). As hypothesized, and paralleling results found for the broader internalizing domain, we also found that daily stressors interacted with 5-HTTLPR genotype to predict both same-day fear symptoms (*χ*^2^(2) = 7.13, *P* <0.05) and same-day distress symptoms (*χ*^2^(2) = 4.90, *P* = 0.08), with SS and SL groups showing stronger stress-symptom associations than L homozygotes (Table 2). These effects of daily stressors and 5-HTTLPR on fear and distress symptoms were stronger when the SS and SL genotypes were grouped and compared to L homozygotes (that is, fear, *P* <0.001; distress, *P* <0.01; Table 3).

### Stress, 5-HTTLPR and next-day internalizing and externalizing symptoms

Consistent with prior research, HLM analyses indicated that stress was not associated with next-day reports of internalizing symptoms (*b* = -0.08, *SE* = 0.08, *P* = 0.32) or externalizing symptoms (*b* = -0.01, *SE* = 0.03, *P* = 0.71). In addition, 5-HTTLPR genotype did not moderate the lagged stress-symptom association for internalizing symptoms (*χ*^2^(2) = 0.76, *P* = 0.68) or externalizing symptoms (*χ*^2^(2) = 2.69, *P* = 0.26).

## Discussion

The validity of the 5-HTTLPR stress sensitivity hypothesis has been challenged by inconsistent results in the literature on life stress, the 5-HTTLPR genotype, and depression. To address this issue, we examined stress exposure and emotional reactivity in ‘high resolution’ using a daily diary methodology. We also evaluated the specificity of 5-HTTLPR G × E effects to depressive symptoms with the use of a novel measure of transdiagnostic emotional phenotypes. Consistent with hypotheses and prior research, we found that daily stress levels were associated with severity of internalizing symptoms, but only for 5-HTTLPR S allele carriers. Going beyond existing work on this topic, however, we also found that this interaction effect extended to both the fear and distress subdomains of internalizing symptoms, suggesting that 5-HTTLPR G × E effects are not unique to depression. Yet, there was only weak support for the hypothesis that stress-induced externalizing behaviors are more common among S allele carriers. These data are in accord with the general hypothesis that variation at 5-HTTLPR plays a role in regulating emotional reactivity to daily life stress.

In line with new dimensional approaches to psychiatric classification, such as the National Institute of Mental Health Research Domain Criteria initiative [23], these results suggest that genetic variation at 5-HTTLPR may influence transdiagnostic stress reactivity mechanisms that contribute to the onset or development of a variety of anxiety and depressive disorders, not just unipolar depression (cross-reference [1,8]). One implication of this transdiagnostic formulation is that studies on 5-HTTLPR could be more efficiently designed by selecting outcomes that are theorized to be common to several categorically defined internalizing syndromes. For instance, in longitudinal G × E studies, 5-HTTLPR may be more closely linked to intermediate phenotypes, such as a latent distress trait, than to the unipolar depression syndrome. We speculate that such an approach may be appropriate for other candidate genes in psychopathology research as well [24].

The present findings also suggest that internalizing risk associated with the S allele can be detected by observing emotional responses to everyday stressors. Through the use of genetically informed daily process studies, it may be possible - even in nonclinical populations - to identify genotypes that confer risk for clinically significant emotional disorders that are provoked by negative life events. Fear and distress responses to daily negative life events may therefore represent useful intermediate phenotypes on the causal pathway from genes to full-blown internalizing disorder.

The present results are consistent with other data showing that the interactive effects of 5-HTTLPR and stress on emotional outcomes are most robust when sophisticated stress assessment methods are used [25]. Indeed, a major strength of the present study was that stress was measured daily, on a within-person basis. Put another way, the degree of stress exposure on a given day was compared to each participant’s average level of stress over the 14-day study period and not to the average level of stress exposure in the full sample. As a result, participants served as their own control when determining the magnitude of day-to-day fluctuations in stress reporting, thereby limiting the potentially confounding effects of state affect or personality traits on self-reported stress exposure.

The relatively modest sample size of the present study (*n* = 104) must be taken into account when evaluating the present results. We reasoned that a test of our hypotheses based on a smaller sample was justified because our theory was based on an existing nomological net of relations between 5-HTTLPR and stress reactivity phenotypes [7]. In other words, the established construct validity of the phenomenon under study was assumed to reduce the danger of false positive findings [26]. Nevertheless, conclusions regarding genetic reactivity to everyday stressors should be considered tentative until large-scale replication projects are available.

Several other limitations should also be noted. First, daily stress exposure, emotions and behaviors were reported at the same time by the same informant; therefore, it was not possible to confirm that the occurrence of stressors preceded the development of symptoms each day. Second, given that fear, distress and externalizing phenotypes have not been investigated previously in within-subjects research, the questionnaires in this study were novel and would benefit from validation in future work. Related, the validity of daily stress assessments for determining genetic differences in stress reactivity has yet to be established in the G × E literature. Future studies should examine whether the same genotype confers risk to both short- and long-term stress reactions in the same sample. Third, our analyses were focused on transitory changes in mood and behavior as a potential intermediate phenotype for emotional disorders; additional research is needed to determine the social and neurobiological processes involved in the activation of more severe and sustained symptomatology. Fourth, we did not correct for multiple testing given strong *a priori* hypotheses and limited sample size, and this analytic decision should be kept in mind when interpreting significance values. Fifth, the prevalence of individual stressor types was relatively low and did not permit analyses to determine whether the magnitude of G × E varied across types of stress. Determining the features of life stress that are most relevant to 5-HTTLPR G × E may ultimately lead to more effective prevention and intervention efforts. Finally, although the 5-HTTLPR G × E effects in our sample were equivalent across the two main ethnic groups, we did not use genetic controls for ethnic heterogeneity (see [27] for potential dangers of ethnic heterogeneity in genetic research).

## Conclusions

The present data demonstrate for the first time that 5-HTTLPR interacts with experiences of daily life stress to predict transdiagnostic internalizing dimensions. Specifically, stress was associated with severity of internalizing symptoms on a day-to-day basis for 5-HTTLPR S allele carriers but not for L allele homozygotes. Additionally, this G × E effect was present across fear and distress dimensions, two subdomains of internalizing symptomatology identified in previous studies of the latent structure of internalizing disorders. These results are consistent with between-subjects research documenting associations of 5-HTTLPR with risk for diverse internalizing syndromes, but extend prior findings by showing that the S allele confers vulnerability to transdiagnostic emotion dysregulation phenotypes, as compared to specific psychiatric disorders. Further research on transdiagnostic emotional phenotypes may help elucidate connections between life stress, candidate genes, and the fundamental components of psychopathology.

## Appendix

Daily fear symptoms

My heart was racing or pounding

I was afraid that I might think or do something bad

I felt self-conscious knowing that others were watching me

I had disturbing thoughts of something bad that happened to me

I felt panicky

Daily distress symptoms

I worried

I felt depressed

I felt discouraged about things

I felt nervous

I felt inadequate

I had little interest in my usual hobbies or activities

Daily externalizing symptoms

I was mean to others

I used drugs (e.g., marijuana) for nonmedical purposes

I broke or threw things

I screamed or yelled

I broke rules at school, work, or elsewhere

I drank too much alcohol or got drunk

I spread rumors or gossiped about someone

I intentionally ignored someone

I lied to get what I wanted

Daily stressful life events

Did not have enough money to do something or buy something

Lost money or something important

Property was damaged or stolen

Was sick or had a medical issue

Did poorly on, or failed, an important exam or major project

Failed to achieve an important school related goal that does not involve GPA

Problems at work (e.g. didn’t get the schedule that you requested, couldn’t find someone to fill in for you)

Problems with co-workers or boss (if different from above)

An event that happened today related to a family member or close friend having a medical or emotional problem

Had an argument/problem with significant other

Had an argument/problem with a friend

Had an argument/problem with family member

Had an argument/problem with a professor, or project group

Fight or argument among social group to which you belong

Was rejected or excluded by others (group, significant other, friend, etc.)

Was criticized by others (project group, significant other, friend, professor, etc.)

## Competing interests

The authors declare that they have no competing interests.

## Authors’ contributions

All authors conceived of the study and participated in its design and coordination. CCC collected the data, performed statistical analyses, and wrote the first draft of the manuscript. GMS and CH edited the manuscript and approved the final version. All authors read and approved the final manuscript.
